# An acute pelvic pain in high-altitude tourist: A case report of ruptured ectopic pregnancy with low β-human chorionic gonadotropin

**DOI:** 10.1097/MD.0000000000046804

**Published:** 2025-12-26

**Authors:** Fang Yuan, Zhan-Cheng Cui, Zhen-Zhen Jiang, Xia-Tian Liu

**Affiliations:** a Department of Ultrasound, Shaoxing People’s Hospital, Shaoxing, Zhejiang, PR China; b Department of Emergency, Da Chai Dan Administrative Commission People’s Hospital, Delingha, Qinghai, PR China; c School of Medicine, Shaoxing University, Shaoxing, Zhejiang, PR China.

**Keywords:** β-hCG, ectopic pregnancy, high-altitude acclimatization, ultrasound

## Abstract

**Rationale:**

High-altitude acclimatization triggers physiological adaptations – such as increased ventilation, heart rate, and red blood cell production – to improve tissue oxygen delivery. These adaptations may obscure typical signs of hemorrhage, complicating the diagnosis of ruptured ectopic pregnancy in patients with low beta-human chorionic gonadotropin (β-hCG) levels.

**Patient concerns:**

A 31-year-old woman developed sudden pelvic pain and mild vaginal bleeding on the third day of high-altitude travel (Qinghai-Tibet Plateau).

**Diagnoses:**

Initial evaluation at a local hospital indicated very low β-hCG and no intrauterine gestational sac, leading to a misdiagnosis of biochemical pregnancy. Hemoglobin stability and absence of red blood cell decline, combined with high-altitude physiological changes, further delayed diagnosis. A transvaginal ultrasound later identified a ruptured right fallopian tube ectopic pregnancy, confirmed surgically.

**Interventions:**

Surgical confirmation.

**Outcomes:**

The case underscores how high-altitude hematologic adaptations (e.g., masked anemia) and low β-hCG levels can mimic benign conditions, complicating timely detection.

**Lessons:**

Ectopic pregnancy should remain a differential diagnosis in high-altitude travelers with abdominal pain, even with low β-hCG and stable hematologic parameters. Clinicians should be vigilant for ectopic pregnancy even with low β-hCG and stable hemoglobin in high-altitude travelers, as acclimatization may mask hemorrhagic signs.

## 1. Introduction

Ectopic pregnancy refers to abnormal implantation of a fertilized ovum out of the intrauterine cavity. The fallopian tube is the most common site. Most cases of tubal ectopic pregnancies that are detected early can be treated successfully either with minimally invasive surgery or with medical management using methotrexate. However, tubal ectopic pregnancy in an unstable situation with symptoms of an ongoing ruptured ectopic mass (such as pelvic pain) or signs of intraperitoneal bleeding is a medical emergency that requires prompt surgical intervention.^[1]^ Upon high-altitude experience, humans acclimate to high altitude through a series of physiological responses among the different systems of the body, such as time-dependent rise in ventilation, heart rate, and redistribution of blood flow that serves to sustain oxygen supply to vital organs.^[2]^ High-altitude acclimatization also stimulates erythropoietin production, leading to increased red blood cell (RBC) count, hemoglobin, and hematocrit.^[3]^ Therefore, the diagnosis of ruptured ectopic pregnancy in patients undergoing high-altitude adaptation is challenging. To the best of our knowledge, this is the first documented case of a patient with atypical ectopic pregnancy symptoms exacerbated by high-altitude acclimatization.

## 2. Case presentation

A 31-year-old female (gravida 2, live 1) from a low altitude presented to our emergency department on July 8 with acute pelvic pain and mild vaginal bleeding on the third day of her high-altitude trip. Her last menstrual period was June 16. She had no previous medical history and was not taking any medication.

**Figure 1.**
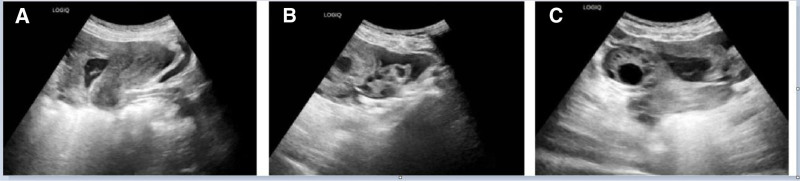
Transabdominal ultrasound characteristics for the first-time examination. (A) Transabdominal ultrasound showed endometrial thickness of 1.1 cm and pelvic effusion depth of 3.0 cm. (B) The image of the left ovary. (C) The image of the right ovary.

**Figure 2.**
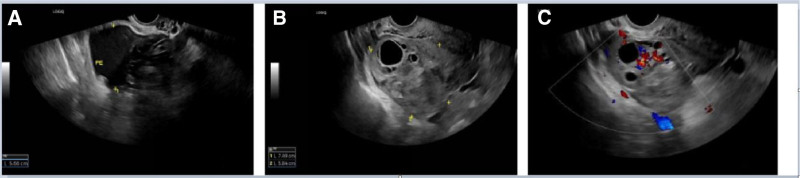
Transvaginal ultrasound characteristics for the second-time examination. (A) Ultrasound images showed that the depth of the pelvic effusion increased to 5.7 cm after 2 hours. (B) A 7.4 cm × 5.8 cm mixed-echo mass with indistinct margins was noted adjacent to the right ovary, with increased pelvic fluid (5.7 cm). (C) Color Doppler image showed that there were blood flow signals within the mass.

**Figure 3.**
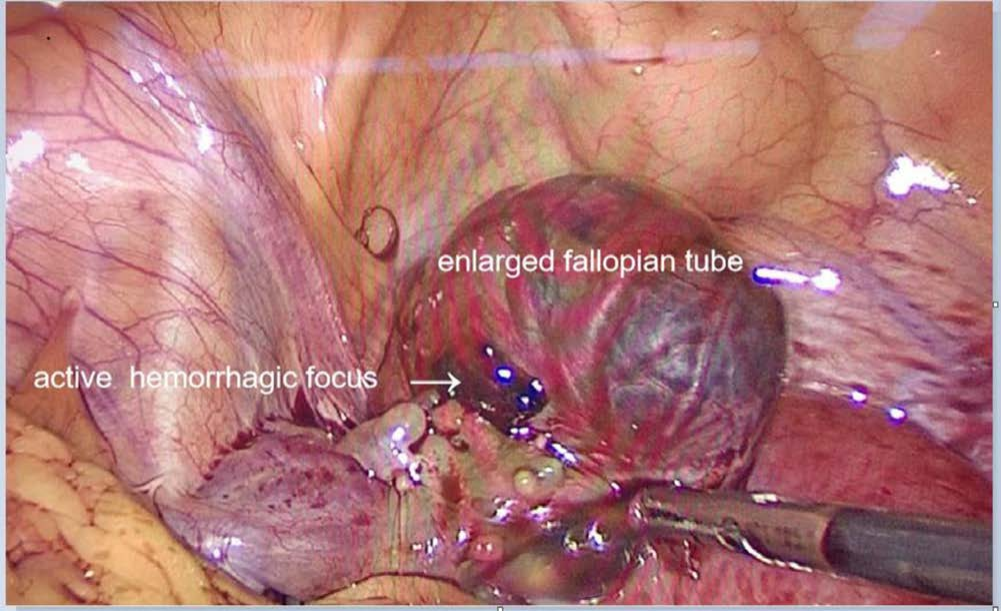
Laparoscopic view showing a 2-cm ampullary rupture of the right fallopian tube with active bleeding and 800 mL hemoperitoneum. The right fallopian tube was enlarged with an active hemorrhagic focus on 1 side.

**Figure 4.**
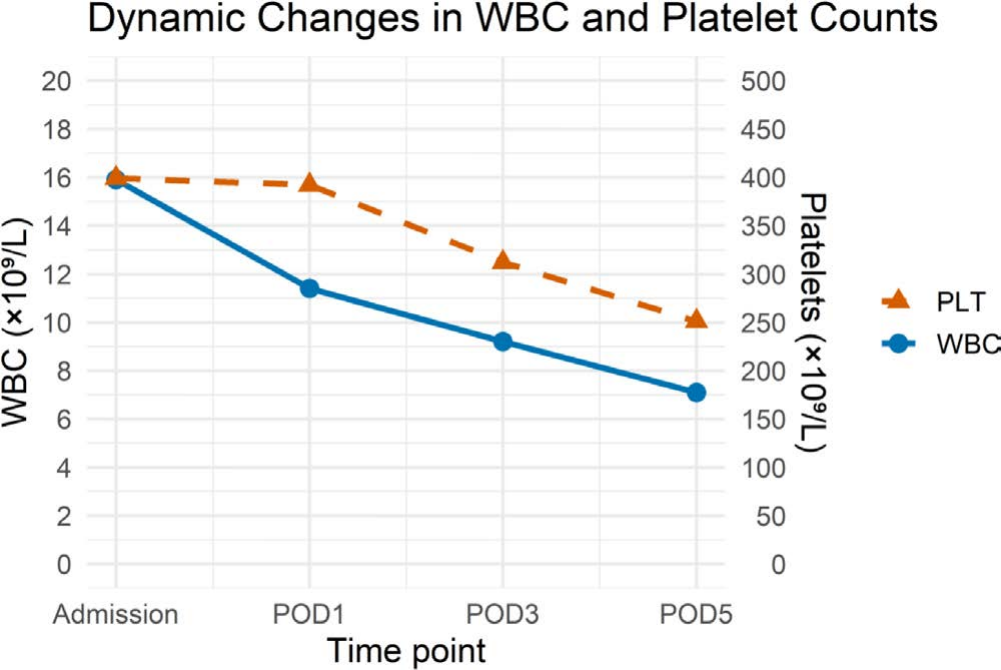
Dynamic changes in WBC and platelet counts over postoperative days 1 to 5. WBC and platelet counts declined in parallel from POD1 to POD5, consistent with resolution of altitude-induced demargination and acute stress response. Values normalized by POD5 (WBC 7.1 × 10⁹/L, platelets 251 × 10⁹/L). PLT = platelet, POD = postoperative day, WBC = white blood cell.

Since she presented with acute distress and was unable to tolerate abdominal palpation and gynecological examination, we conducted hematological examination and transabdominal ultrasound. The laboratory tests (Table 1) showed increased white blood cell (WBC) and neutrophil percentages (NEU), with stable RBC and hemoglobin levels. In particular, transabdominal ultrasound detected pelvic effusion (3.0 cm deep), but no visible intrauterine gestational sac or ectopic pregnancy sac (Fig. 1). Until then, the possibility of inflammation rather than a ruptured ectopic pregnancy has been suggested. Further investigation revealed that the patient had been diagnosed with a biochemical pregnancy in May at a local hospital. Her initial beta-human chorionic gonadotropin (β-hCG) was 33.0 mIU/mL, and no follow-up tests were performed. Her previous menstrual period (PMP) was May 5, and had light bleeding from June 16th, which could have been mistaken for LMP. Given this complex situation, gynecologists and infectious disease specialists were summoned to the emergency department. Within an hour, worsening pain prompted a transvaginal ultrasound scan and quantitative serum β-hCG measurement. A mixed-echo mass (7.4 cm × 5.8 cm) in the right adnexal area with increased pelvic fluid (5.7 cm deep) were visible on sonography and the level of β-hCG was 24.4 mUI/mL (Table 1, Fig. 2). Owing to worsening of the condition and suspicion of a ruptured ectopic pregnancy, the patient was transferred for surgery, confirming a ruptured right fallopian tube ectopic pregnancy with hemorrhage (Fig. 3). Under general anesthesia, laparoscopy revealed 800 mL hemoperitoneum. A 2-cm ampullary rupture of the right tube was identified. Linear salpingotomy was performed with evacuation of the gestational sac; bleeding points were coagulated with bipolar forceps. Postoperatively, the patient made an uneventful recovery. Serial blood counts demonstrated a rapid, parallel decline in WBC and platelet numbers: on postoperative day (POD) 1, WBC was 11.4 × 10⁹/L (neutrophils 76%) and platelets 392 × 10⁹/L; by POD3 these had fallen to 9.2 × 10⁹/L and 312 × 10⁹/L, respectively, and reached 7.1 × 10⁹/L and 251 × 10⁹/L on POD5 (Fig. 4).

**Table 1:** Laboratory tests.

Parameter	Value	Normal ranges
White blood cell	15.9 (×10^9^/L) ↑	3.5–9.5 (×10^9^/L)
Lymphocyte %	10.9 (%)	20–50 (%)
Monocyte %	2.2 (%)	3–10 (%)
Neutrophils %	86.6 (%) ↑	40–75 (%)
Lymphocyte	1.7 (×10^9^/L)	1.1–3.2 (×10^9^/L)
Monocyte	0.35 (×10^9^/L)	0.10–0.60 (×10^9^/L)
Neutrophils	13.8 (×10^9^/L) ↑	1.8–6.3 (×10^9^/L)
Red blood cell	4.8 (×10^12^/L)	3.8–5.1 (×10^12^/L)
Hemoglobin	146 (g/L)	115–150 (g/L)
Hematocrit	42.6 (%)	35–45 (%)
Mean corpuscular volume	89.6 (fL)	82–100 (fL)
Mean corpuscular hemoglobin	31 (pg)	27–34 (pg)
Platelet count	399 (×10^9^/L) ↑	125–350 (×10^9^/L)
Platelet hematocrit	0.37 (%) ↑	0.108–0.282 (%)
β-HCG	24.438 mIU/mL ↑	<5 mIU/mL

β-hCG = beta-human chorionic gonadotropin.

## 3. Discussion

Ectopic pregnancy, a leading cause of maternal mortality in early pregnancy, typically presents with pelvic pain, vaginal bleeding, elevated β-hCG levels, and amenorrhea. Ectopic pregnancy is an obstetric emergency that requires immediate diagnosis and appropriate treatment. Life-threatening complications include tubal rupture and hemoperitoneum.^[4]^ However, this case demonstrates the complexities of diagnosis when an atypical presentation overlaps with physiological changes from altitude acclimatization. In this patient, the initial stable hemoglobin and RBC levels, combined with elevated WBC and NEU %, were misleading. These findings suggest an inflammatory process rather than an acute hemorrhagic event typically observed in ruptured ectopic pregnancies. Moreover, a previous diagnosis of biochemical pregnancy contributed to a diagnostic preconception error, underscoring the challenge of managing patients with complex histories and atypical laboratory results.

Altitude acclimatization is a physiological process that restores oxygen delivery to tissues and promotes oxygen use under high-altitude hypoxia.^[5]^ The transient rise in WBC and platelets shortly after high-altitude ascent is a documented phenomenon, driven primarily by catecholamine-mediated demargination in response to hypoxic stress. This mechanistic understanding is supported by empirical evidence of increased platelet activation and a concomitant systemic endocrine-inflammatory responseunder acute hypobaric hypoxia.^[6–11]^ In a 2023 prospective cohort of 56 lowlanders flown to Lhasa (3658 m), median WBC increased from 6.1 to 9.4 × 10⁹/L on day 2 and normalized by day 7; platelets rose from 230 to 285 × 10⁹/L and returned to baseline after descent.^[12]^ Our patient showed an identical pattern: admission WBC 15.9 × 10⁹/L (NEU % 86.6%) and platelets 399 × 10⁹/L fell to 7.1 × 10⁹/L and 251 × 10⁹/L, respectively, on POD5, confirming reversibility. Studies indicate that the HIF-1 signaling pathway plays a central role in upregulating erythropoietin and altering metabolic processes, which can increase blood cell counts^[5]^ and result in a systemic inflammatory response.^[13]^ Several previous studies have provided evidence that candidate inflammatory mediators are upregulated in peripheral blood during the first few days of acclimatization.^[14,15]^ The increase in RBC production improves oxygen delivery, but may mask signs of internal bleeding, complicating diagnostic interpretation. In this case, these adaptive responses delayed the recognition of the internal bleeding. Additionally, the increase in platelets and WBCs, possibly due to inflammation caused by acclimatization, further complicates the interpretation of the results.

In the past, a low serum β-hCG level has been reassuring for a relatively benign course. According to the American Academy of Family Physicians (AAFP), expectant management can be considered when the patient is hemodynamically stable, the β-hCG level is <1500 mIU/mL and fails to double within 48 hours, and the patient is reliable for follow-up. However, this is not always true. There are a few case reports of tubal ectopic rupture with persistently reduced and low β-hCG levels (Table 2).^[16–19]^ Based on these analyses, it can be concluded that the risk of tubal rupture varies across a broad spectrum of β-hCG levels; thus, β-hCG levels alone do not consistently serve as a reliable indicator for determining whether to pursue surgical, medical, or expectant management. Regrettably, the mixed-echo mass in the right adnexal area was not initially observed on transabdominal sonography. A possible reason may be insufficient bladder filling and interference of intestinal gas, both of which obscure pelvic structure observation. However, transvaginal sonography is free from the interference of gas and has a significantly higher detection rate of ruptured-type ectopic gestation (93.5%) than transabdominal sonography (87.0%; *P* < .05).^[20]^ In summary, ultrasound ultimately proved essential, as sonographic changes over time revealed a significant increase in pelvic fluid and adnexal masses, leading to the diagnosis of ruptured ectopic pregnancy. This emphasizes the value of dynamic imaging in patients with evolving symptoms, particularly when confounding factors such as altitude adaptation and low β-hCG levels are present.

**Table 2:** Summarize characteristics about low β-hCG ruptured ectopic pregnancy in the literature.

Reports	β-hCG (mUI/mL; initial-ruptured)	Symptoms	Previous treatment	Learning points
Kooi et al^[16]^	167–23.7	Increasing right lower quadrant pain, vaginal spotting	Expectant management	Until the time that impending tubal rupture can be judged from a reliable parameter, conservative management is only safe under good surveillance and patient instruction, even in patients with very low and declining serum β-hCG concentrations
Fu et al^[17]^	Below the discriminatory zone-5	Sudden onset of severe abdominal pain, peritoneal signs and hemoperitoneum	Expectant management	Rupture may occur at any time and even with extremely low β-HCG levels. Immediate action should be taken if symptoms develop, and β-hCG levels should be followed to 0
Davis et al^[18]^	585–5	Vaginal spotting and mild lower abdominal pain	Expectant management	Rupture in an expectantly managed ectopic pregnancy can occur when initial β-hCG is low, when it is persistently falling, and even after a long follow-up with a very low level of β-hCG. Serial monitoring of β-hCG in such pregnancies should be continued even after they have fallen to negligible levels
Prabhakaran et al^[19]^	454–636	Lower back pain, cramping pelvic pain, and vaginal spotting	Methotrexate therapy	The risk of tubal rupture varies across a wide range of β-hCG levels. β-hCG is not always a reliable indicator of deferring surgical management for medical management

β-hCG = beta-human chorionic gonadotropin.

This case highlights the importance of considering altitude-related physiological changes in tourists presenting with acute pelvic pain at high altitudes, where ectopic pregnancy should remain differential despite nonclassical presentations. Continuous reevaluation, serial imaging, and awareness of potential biases are crucial for a timely and accurate diagnosis in such complex scenarios.

## 4. Conclusions

Ectopic pregnancies that are not adequately managed are associated with a high mortality rate. This case emphasizes how high-altitude acclimatization affects hematologic markers and complicates the diagnosis of ectopic pregnancy. Clinicians should maintain a high index of suspicion for internal bleeding in high-altitude patients even when the initial blood markers appear stable.

## Author contributions

**Conceptualization:** Zhan-Cheng Cui.

**Data curation:** Fang Yuan.

**Funding acquisition:** Xia-Tian Liu.

**Investigation:** Zhan-Cheng Cui.

**Project administration:** Xia-Tian Liu.

**Supervision:** Zhen-Zhen Jiang.

**Visualization:** Zhen-Zhen Jiang.

**Writing – original draft:** Fang Yuan.

**Writing – review & editing:** Zhen-Zhen Jiang.
